# Supplementary material for: Estimating genetic variance contributed by a quantitative trait locus: A random model approach
Source: PLoS Comput Biol. 2022 Mar 11;18(3):e1009923. doi: 10.1371/journal.pcbi.1009923 (PMC8942241; doi:10.1371/journal.pcbi.1009923)
Supplement: S1 Note — Table A: Estimated parameters for trait KGW from the fixed model, the random model and the Bayesian analysis for bin 725 of the IMF2 rice population. (DOCX) [file pcbi.1009923.s007.docx]

**S7 Note: Bayesian Estimate of QTL Variance ($\sigma_{\alpha}^{2}$)**

The Bayesian estimate of $\sigma_{\alpha}^{2}=\alpha^{2}$ is drastically different from the maximum likelihood estimate of $\sigma_{\alpha}^{2}$ in the situation of regression analysis. The reason is that the variance is defined and estimated from a “single group level.” A good Bayesian estimate of a variance component needs at least three groups [1-3]. For example, if the group level is three, the variance is defined as

$$\sigma_{\alpha}^{2}=\frac{1}{3}(\alpha_{1}^{2}+\alpha_{2}^{2}+\alpha_{3}^{2})$$

We just do not have enough information to estimate $\sigma_{\alpha}^{2}$ if we only have one group level. It is commonly believed that the maximum likelihood estimate of a parameter is equivalent to the Bayesian estimate if the prior of the parameter is uniform. Unfortunately, this is not the case. We now performed Bayesian analysis for the KGW trait of the hybrid rice population. We investigated five different priors listed below,

(1) Uniform prior on $\sigma_{\alpha}^{2}$, i.e., $p(\sigma_{\alpha}^{2})\propto1$ and the log prior density is 0. In the PROC MCMC software package, such a prior is coded as

prior $\sigma_{\alpha}^{2}$ ~ general(**0**);

(2) Uniform prior on $\sigma_{\alpha}$, which is equivalent to $p(\sigma_{\alpha}^{2})\propto\sigma_{\alpha}^{-1}$. In PROC MCMC, this is coded as

prior $\sigma_{\alpha}^{2}$ ~ general(-$\log(\sigma_{\alpha})$);

(3) Uniform prior on $\log(\sigma_{\alpha})$, equivalent to $p(\sigma_{\alpha}^{2})\propto\sigma_{\alpha}^{-2}$. In PROC MCMC, this is coded as

prior $\sigma_{\alpha}^{2}$ ~ general(-$\log(\sigma_{\alpha}^{2})$);

(4) Uniform prior on $\log(\sigma_{\alpha})$, equivalent to $p(\sigma_{\alpha})\propto\sigma_{\alpha}^{-1}$. In PROC MCMC, this is coded as

prior $\sigma_{\alpha}$ ~ general(-$\log(\sigma_{\alpha})$);

(5) Half-Cauchy on $\sigma_{b}$, the prior density is

$$p(\sigma_{\alpha})=\left\{\begin{matrix} \frac{2}{\pi\theta}\times\frac{1}{1+\sigma_{\alpha}^{2}/\theta^{2}} \\ 0 \end{matrix}\right.\begin{matrix} \\ \end{matrix}\begin{matrix} \sigma_{\alpha}\geq0 \\ \text{otherwise} \end{matrix}$$

where $\theta>0$. In PROC MCMC, this is coded as

prior $\sigma_{\alpha}$ ~ general(log(2)-log($\pi$)-log($\theta$)-log(1+$\sigma_{\alpha}^{2}$/$\theta^{2}$));

The MCMC procedure in SAS was used to implement the Bayesian method for parameter estimation. Since coding PROC MCMC for the polygenic model using the marker-inferred kinship matrix is very difficult, we only investigated the simple model without the polygenic background control. The initial values of the intercept and QTL effect were set at 0. The initial values of the residual variance and the QTL variance were set at 1. The burn in period was 1000. The total number of MCMC iterations after burn in was 100,000. The thin rate was 100. Therefore, the posterior sample contained $100000/100=1000$ observations. The PROC MCMC code is enclosed below (**Code A**), which takes the half-Cauchy prior on the QTL standard deviation ($\sigma_{\alpha}$). **Table A** shows the results of Bayesian estimates of parameters in comparison with the estimates of the restricted maximum likelihood methods.

**Code A** SAS code to read the data, perform the MCMC analysis and write the posterior sample to an external excel data sheet.

**proc** **import** datafile="Data1-phegen725.xlsx" out=phe dbms=xlsx replace;

**run**;

ods graphics on;

**proc** **mcmc** data=phe outpost=postsample nmc=**100000**

thin=**100** seed=**246810** nbi=**1000** ntu=**3000**

monitor=(b0 b1 sigma2 sigmab)

diag=Geweke(f1=**0.25** f2=**0.25**);

ods select PostSumInt Geweke TADpanel;

parms b0 **0**;

parms b1 **0**;

parms sigma2 **1**;

parms sigmab **1**;

begincnst;

tau=**1e-10**;

omega=**1e-10**;

pi=**3.1415926**;

a=**100**;

endcnst;

prior b0 ~ normal(mean = **0**, var = **1e10**);

prior b1 ~ normal(mean = **0**, sd = sigmab);

prior sigma2 ~ sichisq(tau,omega);

prior sigmab ~ general(-log(**2**)-log(pi)-log(a)-log(**1**+sigmab****2**/a****2**));

mu = b0+b1*z;

model y ~ normal(mean = mu, var = sigma2);

**run**;

ods graphics off;

**proc** **export** data=postsample outfile="prior-5.csv" dbms=csv replace;

**run**;

**proc** **univariate** data=postsample;

var sigmab;

histogram sigmab;

**run**;

**Table A** Estimated parameters for trait KGW from the fixed model, the random model and the Bayesian analysis for bin 725 of the IMF2 rice population ($\sigma_{QTL}^{2}=\sigma_{\alpha}^{2}=\alpha^{2}$)

| Method | $\hat{\alpha}$ | $\hat{\sigma}^{2}$ | $\hat{\sigma}_{\alpha}^{2}$ |
| --- | --- | --- | --- |
| REML (Fixed model approach) | 0.6078 (0.09963) | 2.7496 | 0.3595 |
| REML (Random model approach) | 0.5915 (0.09828) | 2.7496 | 0.3595 |
| Bayes (Uniform on $\sigma_{\alpha}^{2}$,$p(\sigma_{\alpha}^{2})\propto1$) | 0.6046 (0.0974) | 2.7712 | 3.0E13 |
| Bayes (Uniform on $\sigma_{\alpha}$,$p(\sigma_{\alpha}^{2})\propto\sigma_{\alpha}^{-1}$) | 0.6097 (0.1012) | 2.7709 | 1.0E07 |
| Bayes (Uniform on $\log(\sigma_{\alpha})$,$p(\sigma_{\alpha}^{2})\propto\sigma_{\alpha}^{-2}$ | 0.5924 (0.1032) | 2.7692 | 7.50 |
| Bayes (Uniform on $\log(\sigma_{\alpha})$, $p(\sigma_{\alpha})\propto\sigma_{\alpha}^{-1}$ | 0.5924 (0.0979) | 2.7665 | 25.0 |
| Bayes (Half-Cauchy on $\sigma_{\alpha}$, $\theta=1$) | 0.5943 (0.0997) | 2.7675 | 1.0 |
| Bayes (Half-Cauchy on $\sigma_{\alpha}$, $\theta=10$) | 0.6019 (0.1010) | 2.7681 | 16.0 |
| Bayes (Half-Cauchy on $\sigma_{\alpha}$, $\theta=100$) | 0.5998 (0.0994) | 2.7675 | 400.0 |

**References**

1. Gelman A. Prior distributions for variance parameters in hierarchical models (comment on article by Browne and Draper). bayesian analysis. 2006;1(3):515-34. doi: 10.1214/06-BA117A.

2. Gelman A, Carlin JB, Stern HS, Rubin DB. Bayesian Data Analysis. New York: Chapman & Hall/CRC; 2004.

3. Gelman A, Jakulin A, Pittau MG, Su Y-S. A weakly informative default prior distribution for logistic and other regression models. The Annals of Applied Statistics. 2008;2(4):1360-83.
