# Supplementary material for: Estimating genetic variance contributed by a quantitative trait locus: A random model approach
Source: PLoS Comput Biol. 2022 Mar 11;18(3):e1009923. doi: 10.1371/journal.pcbi.1009923 (PMC8942241; doi:10.1371/journal.pcbi.1009923)
Supplement: Attachment — Submitted filename: Response-9-24-2021.docx [file pcbi.1009923.s008.docx]

PCOMPBIOL-D-21-01214

**Response to Editor’s Comment**

Dr. Jian Ma was the editor to evaluate the initial submission of the manuscript. The decision of the initial submission was “return without review.” The main reason was that the manuscript did not have an application to real biological problems. I had several email exchanges with Professor Ma. He suggested that if I add a biological application to the manuscript, it may be sent out for further review. Following his suggestion, we added an application to QTL mapping in mice. Two additional figures were added corresponding to the real life application. We hope that the manuscript is sufficient to warrant a peer review. Thank you for reconsideration of this resubmission.

Best regards,

Shizhong Xu
