# Supplementary material for: Estimating genetic variance contributed by a quantitative trait locus: A random model approach
Source: PLoS Comput Biol. 2022 Mar 11;18(3):e1009923. doi: 10.1371/journal.pcbi.1009923 (PMC8942241; doi:10.1371/journal.pcbi.1009923)
Supplement: Attachment — Submitted filename: review for PCOMPBIOL.docx [file pcbi.1009923.s009.docx]

This manuscript proposes a random-effect model approach to estimating the QTL variance.

The method reformulates the QTL model by treating the QTL effect as random and directly estimate the QTL variance (as a variance component) or adjust the bias by taking into account the error of the estimated QTL effect. A moment method of estimation has been proposed to correct the bias. The method has been validated via Monte Carlo simulation studies. The method has been applied to QTL mapping for the 10-week-body-weight trait from an F2 mouse population. The manuscript was well written, and developed a novel method that can be applied to many real data sets. I evaluate the work as a useful contribution and can be published. I have two comments.

1. The manuscript includes too many equations and derivations, some of which are easily derived and standard. I recommend simplify the mathematical presentation and thus improve readable.
2. The proposed random-effect assumes that the QTL effects follow a normal prior. What is the prior on the prior variance? You use an uniform prior. However, it results in a estimation towards zero. Gelman et al. Bayesian Data Analysis (Chapter 5) suggests weakly informative prior, which can solve the problem.
