# Supplementary material for: Estimating genetic variance contributed by a quantitative trait locus: A random model approach
Source: PLoS Comput Biol. 2022 Mar 11;18(3):e1009923. doi: 10.1371/journal.pcbi.1009923 (PMC8942241; doi:10.1371/journal.pcbi.1009923)
Supplement: Attachment — Submitted filename: Response.docx [file pcbi.1009923.s010.docx]

**Response to Reviewer #1**

C: Reviewer’s Comment

R: Authors’ Response

C0: The method reformulates the QTL model by treating the QTL effect as random and directly estimate the QTL variance (as a variance component) or adjust the bias by taking into account the error of the estimated QTL effect. A moment method of estimation has been proposed to correct the bias. The method has been validated via Monte Carlo simulation studies. The method has been applied to QTL mapping for the 10-week-body-weight trait from an F2 mouse population. The manuscript was well written, and developed a novel method that can be applied to many real data sets. I evaluate the work as a useful contribution and can be published.

R0: Your summary is very accurate and thank you.

C1: The manuscript includes too many equations and derivations, some of which are easily derived and standard. I recommend simplify the mathematical presentation and thus improve readable.

R1: We have deleted most of the derivations to shorten the manuscript in the revised manuscript. In fact, we deliberately provided the derivations in the initial submission to help reviewers review the manuscript. We had planned to delete the derivations after the first submission anyway.

C2: The proposed random-effect assumes that the QTL effects follow a normal prior. What is the prior on the prior variance? You use a uniform prior. However, it results in a estimation towards zero. Gelman et al. Bayesian Data Analysis (Chapter 5) suggests weakly informative prior, which can solve the problem.

R2: Our method of estimation for the QTL variance is the maximum likelihood method. The QTL variance can also be estimated from the Bayesian method. We performed Bayesian analysis using uninformative priors and weakly informative priors for the working example data of hybrid rice. The results are shown in Supplementary Table S1. We found that the Bayesian estimate of QTL variance highly depends on the prior distribution of the QTL variance. The maximum likelihood estimate is not the Bayesian estimate with uniform prior of the QTL variance. We thought it was but the result said no. Bayesian estimates of the QTL variance using a wide range of uninformative priors and weakly informative prior are very large compared with the ML estimate. We realized that the Bayesian method cannot be applied to the prior variance of a regression coefficient (with a single group level). Gelman (2016) stated that when the group level is less than 3, there is no sufficient information to estimate the variance using the Bayesian method. We added one paragraph to discuss the Bayesian analysis. We also added Supplementary Note S3 and Supplementary Table S1 to provide detailed information about the Bayesian analysis. Below is the added paragraph of Bayesian analysis to the revised manuscript: “Variance components may also be estimated via the Bayesian method by assigning a prior distribution to each variance components. The Bayesian estimate of $\sigma_{\alpha}^{2}=\alpha^{2}$ is drastically different from the maximum likelihood estimate of $\sigma_{\alpha}^{2}$ in the situation of regression analysis. The reason is that the variance is defined and estimated from a “single group level.” A good Bayesian estimate of a variance component needs at least three group levels (Gelman et al. 2004; Gelman 2006; Gelman et al. 2008). The MCMC procedure in SAS was used to implement the Bayesian method for parameter estimation. Since coding PROC MCMC for the polygenic model using the marker-inferred kinship matrix is very difficult, we only investigated the simple model without the polygenic background control. Five different prior distributions were investigated, including the uniform prior on $\sigma_{\alpha}^{2}$ and a weakly informative half-Cauchy prior on $\sigma_{\alpha}$. Please see **Supplementary Note S3** for a complete list of prior distributions investigated in this project. **Supplementary** **Table S1** shows the results of Bayesian estimates of parameters in comparison with the estimates of the restricted maximum likelihood methods for the hybrid rice data (data of the working example). The estimated QTL effects and residual variances across a range of prior distributions are much the same compared with the estimates from the restricted maximum likelihood methods. However, the Bayesian estimates of the QTL variance are drastically different from the REML estimate and the differences are highly dependent of the prior distributions. Therefore, the proposed MM and REML estimate of the QTL variance under one group level may be the only option for estimating QTL heritability.”

**Response to Reviewer #2**

C: Reviewer’s Comment

R: Authors’ Response

C0: This manuscript presents an old but important question in QTL mapping and GWAS; i.e., how to correct biased genetic variance explained by a single QTL. The manuscript was well written and results are scientifically sound.

R0: Thank you for your support.

C1: In this reviewer's opinion, mapping and estimating a single QTL is not meaningful in practical breeding schemes. As claimed by the authors, a small-effect but statistically significant QTL is not useful in practice. Yet, such QTLs are very commonly detected in plant, animal and human genetic studies. According to a recent study, an insignificant locus by statistical testing is not necessarily insignificant on its merit, rather its effect is compromised by negative regulators (Wang et al. 2021). I believe that an in-depth discussion on this issue is crucial for strengthening this manuscript's quality and impact.

Wang HJ, et al. (2021) Modeling genome-wide by environment interactions through omnigenic interactome networks. Cell Reports 35: 109114.

R1: We added the following paragraph to the discussion to address this comment: “A statistically significant QTL does not mean that it is significant biologically if the QTL contributes a very small proportion of the trait variance. However, a large QTL of biologically significance may not be significant statistically. The lack of power to detect such a QTL is primarily due to interactions of the QTL with other QTLs in linkage disequilibrium. For example, if two QTLs are in high LD but one is an antagonist of the other, neither one may be detected because the effect of one locus would be cancelled by the other. A multiple locus model may improve the detection of both loci. More importantly, each locus may act as a member in a genetic network that consists of many loci (Wang et al. 2021). An individual locus may not be detected alone but is detectable collectively as a network.”

**Response to Reviewer #3**

C: Reviewer’s Comment

R: Authors’ Response

C0: The paper tries to address bias in h2 estimate due to non Beavis effect theoretically and empirically. However, their results suggest that such bias is negligible as long as n is not too small. Given that the Beavis effects over dominate non Beavis ones, practically, it is thus important to simultaneously correct both for studies with very small n, which should be properly addressed. Otherwise, this research has very limited practical usages.

R0: It is true that the study may have limited practical usages. However, it contributes fundamentally to quantitative genetics theory. It also clarifies decades long conceptual confusing in QTL mapping and GWAS. We added two paragraphs to discuss the fundamental contributions of the study. The two paragraphs are shown below:

“The current study contributes more to the quantitative genetics theory than to practical data analysis. Typical QTL mapping and GWAS models include QTL effects as fixed effects while the sizes of QTLs are reported as QTL variances or QTL heritability. The concept of variance does not go well with a fixed effect. It is the random effect that involves a variance. This conceptual relationship has been confused in the QTL mapping community for over three decades. This study has clarified this fundamental relationship.”

“Another fundamental contribution of this study to statistics is the “randomized fixed model approach” to estimating variances. If the number of levels of a random effect is small, this randomized fixed model can be used to estimate the variance associated with the random effect. We estimate the fixed effect as the best linear unbiased estimate (BLUE) and then convert the estimated fixed effect into a variance, like we presented in equation for the estimated QTL variance. A single regression coefficient is considered as just one level of a random effect. For multiple levels of a random effect, say $\alpha=\left[\begin{matrix} \alpha_{1} & \alpha_{2} \end{matrix}\right]^{T}$, and if each level of the random effect follows the same distribution, say $\alpha_{k}\sim N(0,\sigma_{\alpha}^{2})$ for $k=1,2$, the randomized fixed model approach to estimating $\sigma_{\alpha}^{2}$ is a simple extension of the MM estimate, as shown below,

$$\hat{\sigma}_{\alpha}^{2}=\frac{1}{2}tr\left[\hat{\alpha}\hat{\alpha}^{T}-\mathrm{var}(\hat{\alpha}|\alpha)\right]=\frac{1}{2}\sum_{k=1}^{2} \left[\hat{\alpha}_{k}^{2}-\mathrm{var}(\hat{\alpha}_{k}|\alpha_{k})\right]$$

where $\mathrm{var}(\hat{\alpha}|\alpha)$ is a $2\times2$ variance matrix. In quantitative genetics, the four epistatic effects per pair of loci (additive × additive, additive × dominance, dominance × additive and dominance × dominance) may be modeled as a random effect with four group levels. Each level of the epistatic effects follows the same normal distribution denoted by $N(0,\sigma_{\text{EP}}^{2})$. The epistatic variance ($\sigma_{\text{EP}}^{2}$) may be easily estimated using equation with the 2 levels in the formula substituted by 4 levels of the epistatic model. The original random (or mixed) model methodology does not have an explicit estimate of a variance component for the ML and REML methods. The randomized fixed model provides an explicit solution. As a result, this new method is much like the Type3 method of the MIXED procedure in SAS, but it is the QTL mapping version of the Type3 method.”

Other concerns:

C1: Not agree with the comments made between lines 277-282. The variance in (10) is a conditional variance of y given X and Z. The conditional variance of y given X only will depend on Z, so does the marginal variance of y.

R1: You are absolutely right. If you treat $\alpha$ as a fixed effect, the variance of y would not include Z because this term is included in the expectation. This is the very point we are trying to make. The variance of y conditional on X only was given in equation (11) in the original submission and is equation (9) in the revised manuscript. This is the correct variance because the variance depends on Z now.

C2: Equations (16)-(18) are very confusing. So are equations (18) and (19), and it is not clear what is the difference between the two hat estimates of alpha in these two equations.

R2: This part has been deleted in the revised manuscript. You can read the track-change version of the manuscript to see how much we have changed to accommodate your comments.

C3: Section 2 is very wordy and can be significantly shortened. The authors should only present the models and derive the biases concisely without lengthy explanations.

R3: Following your recommendation, we have substantially shortened the method section. You can read the track-change version of the manuscript to see how much we have changed the manuscript to accommodate your comments.
